# Seasonal influence on miRNA expression dynamics of extracellular vesicles in equine follicular fluid

**DOI:** 10.1186/s40104-024-01097-2

**Published:** 2024-10-09

**Authors:** Jean M. Feugang, Ahmed Gad, Nico G. Menjivar, Ghassan M. Ishak, Samuel Gebremedhn, Melba O. Gastal, Notsile H. Dlamini, Radek Prochazka, Eduardo L. Gastal, Dawit Tesfaye

**Affiliations:** 1 Department of Animal and Dairy Sciences, Mississippi State University, Mississippi State, MS 39762 USA; 2 Animal Reproduction and Biotechnology Laboratory (ARBL), Department of Biomedical Sciences, College of Veterinary Medicine and Biomedical Sciences, Colorado State University, Fort Collins, CO 80523 USA; 3 Department of Animal Production, Faculty of Agriculture, Cairo University, Giza, 12613 Egypt; 4 Department of Surgery and Obstetrics, College of Veterinary Medicine, University of Baghdad, Baghdad, 10011 Iraq; 5 Animal Science, School of Agricultural Sciences, Southern Illinois University, Carbondale, IL 62901 USA; 6 J.R. Simplot Company, Kuna, ID 83634 USA; 7 Institute of Animal Physiology and Genetics, Czech Academy of Sciences, Liběchov, 27721 Czech Republic

**Keywords:** Extracellular vesicle, Follicle growth, Follicular fluid, Horse, Mare, Ovulation, Pre-ovulation, Seasonal breeding

## Abstract

**Background:**

Ovarian follicular fluid (FF) is a dynamic environment that changes with the seasons, affecting follicle development, ovulation, and oocyte quality. Cells in the follicles release tiny particles called extracellular vesicles (EVs) containing vital regulatory molecules, such as microRNAs (miRNAs). These miRNAs are pivotal in facilitating communication within the follicles through diverse signaling and information transfer forms. EV-coupled miRNA signaling is implicated to be associated with ovarian function, follicle and oocyte growth and response to various environmental insults. Herein, we investigated how seasonal variations directly influence the ovulatory and anovulatory states of ovarian follicles and how are they associated with follicular fluid EV-coupled miRNA dynamics in horses.

**Results:**

Ultrasonographic monitoring and follicular fluid aspiration of preovulatory follicles in horses during the anovulatory (spring: non-breeding) and ovulatory (spring, summer, and fall: breeding) seasons and subsequent EV isolation and miRNA profiling identified significant variation in EV-miRNA cargo content. We identified 97 miRNAs with differential expression among the groups and specific clusters of miRNAs involved in the spring transition (miR-149, -200b, -206, -221, -328, and -615) and peak breeding period (including miR-143, -192, -451, -302b, -100, and let-7c). Bioinformatic analyses showed enrichments in various biological functions, e.g., transcription factor activity, transcription and transcription regulation, nucleic acid binding, sequence-specific DNA binding, p53 signaling, and post-translational modifications. Cluster analyses revealed distinct sets of significantly up- and down-regulated miRNAs associated with spring anovulatory (Cluster 1) and summer ovulation–the peak breeding season (Clusters 4 and 6).

**Conclusions:**

The findings from the current study shed light on the dynamics of FF-EV-coupled miRNAs in relation to equine ovulatory and anovulatory seasons, and their roles in understanding the mechanisms involved in seasonal shifts and ovulation during the breeding season warrant further investigation.

**Supplementary Information:**

The online version contains supplementary material available at 10.1186/s40104-024-01097-2.

## Introduction

Folliculogenesis is a well-orchestrated regulatory process involving autocrine, paracrine, and endocrine signaling steadily influenced by intrinsic and extrinsic factors in the female body [1–3]. Seasonal variations in temperature extremes, daylight duration, and relative humidity are among the many extrinsic factors that disrupt intrafollicular molecular interactions, leading to impaired fertility in livestock species [1, 4, 5]. Numerous studies have reported the negative impacts of seasonal variation at the cellular (e.g., cell proliferation and apoptosis) and endocrine (e.g., increased production of steroids) levels, affecting tissue organization (e.g., reduction in follicle diameter and luteolytic failure) in farm animals [6–8]. Consistent with these observations, in vitro studies have demonstrated such adverse effects on ovarian follicle growth, oocyte maturation, and embryo production [9–11]. Understanding how environmental factors affect the reproductive capabilities of animals like horses can help improve animal husbandry practices.

The mare is considered a seasonal breeder and is often studied to understand potential changes in fertility during different seasons of the year [1, 12]. The mare typically experiences a major ovulatory wave every 21 to 24 d. During this time, one large follicle of the wave, typically at least 28 mm in diameter, becomes the dominant follicle [13, 14], eventually growing to preovulatory size (around 30–45 mm in diameter) and ovulating in the spring (SOV), summer (SUM), and/or fall (FOV) ovulatory seasons. Meanwhile, homologous dominant follicles regress during the non-breeding seasons, such as the transient spring (SAN**)** and fall anovulatory seasons. The mare enters deep anestrus during the winter with follicles less than 20 mm in diameter [1, 15, 16]. Previous research suggests that during anovulatory seasons and low ovarian activity, the difference in ovarian activities between small and large follicles influences the potential for exogenous gonadotropin-releasing hormone to induce ovulation [1, 17]. These findings underscore the importance of studying preovulatory follicles in the context of seasonal variations, as it has the potential to significantly enhance our understanding and thereby improve female fertility in seasonally inactive species.

Various studies have demonstrated continuous changes in ovarian activity of non-pregnant mares throughout the year, with periods of high activity during the summer, low activity during the winter, and irregular and interspaced activity during the spring and fall transitional seasons [1, 18, 19]. Additionally, aberrant blood flow, hormonal levels, and gene expression changes have previously been observed among follicles from different seasons [19–21]. Recent studies in horses focusing on the effects of seasons demonstrated further aberrations in the gene expression profiles of follicular cells [22], as well as the proteomic profiles [23, 24] and miRNA signatures [25] from follicular fluid (FF) and follicular fluid-derived extracellular vesicles (FF-EVs), respectively. The miRNA, the small noncoding RNA molecules of approximately 22 nucleotides long, are expressed in the ovary to regulate various aspects of follicle growth post-transcriptionally. This includes miR-224, miR-378, and miR-383 in follicle growth, miR-34 in atresia, miR-21 in ovulation, miR-175p and let-7b in angiogenesis, and miR-23a, miR-92, and miR-143 in steroidogenesis [26, 27]. The detection of miRNAs encapsulated in EVs in the ovarian FF of livestock such as cows, pigs, sheep, and mares and their profiles were associated with long-term effects during in vitro fertilization [28]. Research also shows that these miRNAs have effects during heat stress [29, 30] and seasonal changes [31]. Together, these studies conclude that season-specific factors comprising the FF may inherently affect follicle function and oocyte maturation, and the understanding of miRNA dynamics in FF-EVs provides insights into reproductive health and potential therapies.

This study aims to explore the miRNAs associated with equine FF-EVs, which are small lipid-enclosed particles released by various cell types and classified as exosomes (30–130 nm) and microvesicles (50–1,000 nm). These particles are essential in cell-to-cell communication and transferring genetic information within the follicular environment [32, 33]. They transport and shuttle regulatory molecules such as lipids, proteins, and small RNAs to the target cells in a timely delivery system [34, 35]. A recent study observed changes in the miRNA profiles of bovine FF-EVs collected during summer and winter, suggesting the possible role of EV-coupled miRNAs in regulating ovarian response to environmental variations and controlling follicular activity [31]. Here, we hypothesize that the miRNA content of equine FF-EVs varies depending on the ovulatory and anovulatory seasons and influences follicular activity during seasonal variations, which can impact mares’ fertility. Our study aims to investigate the expression and dynamics of the equine FF-EV miRNA profiles in preovulatory follicles during ovulatory (SOV, SUM, and FOV) and anovulatory (SAN) seasons.

## Materials and methods

See Additional file 1 for further details.

### Animals, transrectal ultrasonography and seasonal grouping

The study used 19 multiparous Quarter Horse mares, weighing between 400 and 600 kg and aged 8 to 14 years, grazed freely in the northern hemisphere (latitude, 37.7^o^ N) under natural light conditions with unlimited access to fresh water and trace-mineralized salt. During the non-ovulatory season (SAN) and ovulatory seasons (SOV, SUM, and FOV), transvaginal ultrasound-guided ablations of all ovarian follicles were carried out 10–11 d after the ovulation of the previous cycle to induce a new follicular wave [36]. Then, daily ultrasonographic tracking was performed until the dominant follicle reached a preovulatory diameter. Only animals showing continuous ultrasonographic follicle growth for at least three consecutive days, uterine edema (estrus-like), and the absence of a corpus luteum at the time of FF aspiration were used for sample collection.

### Follicular-fluid sample collection

All mares were adequately prepared and sedated before follicular ablation and FF sampling procedures, as described in previous studies [37, 38]. Preovulatory follicles with a diameter of 30–35 mm were aspirated into sterile vials during specific seasons: SAN (March), SOV (April and May), SUM (June to July), and FOV (September). Following the abovementioned restrictions, only 6 to 12 mares per seasonal group were used, resulting in 42 clear FF samples collected throughout the year. These FF samples were promptly centrifuged (1,600 × *g* for 10 min at 4 °C), and the supernatants were then subjected to subsequent centrifugation (3,200 × *g* for 15 min at 4 °C) and stored at −80 °C for downstream analysis.

### Isolation of extracellular vesicles from follicular fluid

The experiment combined two to three FF samples of individual mares (0.5 mL) to create four biological replicates (1.0–1.5 mL/replicate) for isolating extracellular vesicles (EVs). The FF samples underwent a series of centrifugation steps to remove cells (500 × *g* for 5 min) and debris (4,000 × *g* for 5 min) and were then filtered (0.22 µm) to exclude large particles. The EVs were then isolated using centrifugation (25,000 × *g* for 30 min at 4 °C) and ultracentrifugation (120,000 × *g* for 70 min at 4 °C) procedures. Finally, the isolated EVs were washed, resuspended in PBS, and stored at −80 °C for further analysis.

### Morphological and molecular characterization of FF-EVs

*Specific proteins of frozen-thawed EVs* were analyzed using the JESS Simple Western™ instrument (ProteinSimple^®^, Bio-Techne, Minneapolis, MN, USA). Proteins CD81, FLOT-1, and TSG101 were checked, while the absence of cytochrome C (CYCS) was ensured. Protein lysate (10 µL) was extracted using RIPA lysis buffer and run under JESS’s Assay Module for Protein Normalization (AM-PN001). The analysis used 25 capillary cartridges and specific antibodies, followed by detection and analysis using Compass for Simple Western software. *The size distribution and concentration of EVs* were measured using the Zetaview Particle Metrix (Particle Metrix, Germany). EV samples were diluted (1,000×) in sterile PBS and analyzed using the ZetaView Laser scattering microscope with an LM14C laser. For each sample, 11 independent video measurements were recorded at 11 independent positions, and video files were analyzed using ZetaView software version 8.05.12. *The morphology of EVs* was analyzed using a transmission electron microscope (TEM) according to the methods previously reported [39]. Purified EVs were placed on grids, stained, and viewed using an FEI/TFS Tecnai T12 Spirit TEM. All relevant data have been submitted to the EV-TRACK knowledgebase (https://evtrack.org) with the EV-TRACK ID EV231010 [40].

### Total RNA extraction, library preparation, and sequencing

The EV samples from different experimental groups were used to isolate total RNA, including miRNAs, using a Norgen Exosomal RNA Isolation kit (Norgen, Canada). Genomic DNA contaminants were eliminated using on-column DNA digestion, and RNA concentration and size distribution were assessed using an Agilent 2100 Bioanalyzer (Agilent Technologies, Santa Clara, CA, USA). Small-RNA libraries were prepared for next-generation sequencing with Illumina's TruSeq Small RNA Library Prep Kit. Library quantity and quality were assessed using a Qubit 2.0 Fluorometer and an Agilent 2100 Bioanalyzer. The libraries were then sequenced using a NovaSeq6000 instrument (Illumina, Inc., San Diego, CA, USA), as single-end reads (50 bases).

### Small RNAseq data analysis

The FASTQ files for each sample were generated using the bcl2fastq software. Data analysis was performed using CLC Genomics Workbench version 21. Raw sequencing reads were trimmed based on quality score (Q-score > 30), ambiguous nucleotides (≤ 2 nucleotides), read length (≥ 15 nucleotides), and removal of adapter sequences. The reads were mapped to the equine reference genome (EquCab3.0) and annotated against equine precursor and mature miRNAs (miBase database, release 22). Raw expression data were normalized using the TMM normalization method [41] and presented as TMM-adjusted Counts Per Million (CPM). MiRNAs exhibiting a fold change (FC) ≥ 2 and a *P*‐adjusted value (FDR) < 0.05 [42] were considered differentially expressed (DE). The raw FASTQ and processed CSV files have been deposited in the NCBI’s Gene Expression Omnibus (GEO) under accession number GSE249220.

### Cluster analysis, target gene prediction, and ontological classification

The expressed miRNAs were grouped into different clusters based on their expression profiles during different seasons using the Mfuzz Bioconductor package [43]. The genes targeted by the DE-miRNAs were identified using the human homologous miRNAs in the miRWalk database [44]. Validated target genes from miRTarBase (version 7.0) and standard target gene predictions by TargetScan (version 7.1) and miRDB (release 5.0) within miRWalk were chosen for pathway analysis using the DAVID bioinformatics web tool (https://david.ncifcrf.gov/) and the KEGG pathway database [45]. Furthermore, a network of all DE-miRNAs from different comparisons was created using Cytoscape [46].

## Results

### Seasonal alterations dynamically impact the intrafollicular secretion of EVs

**Fig. 1:**
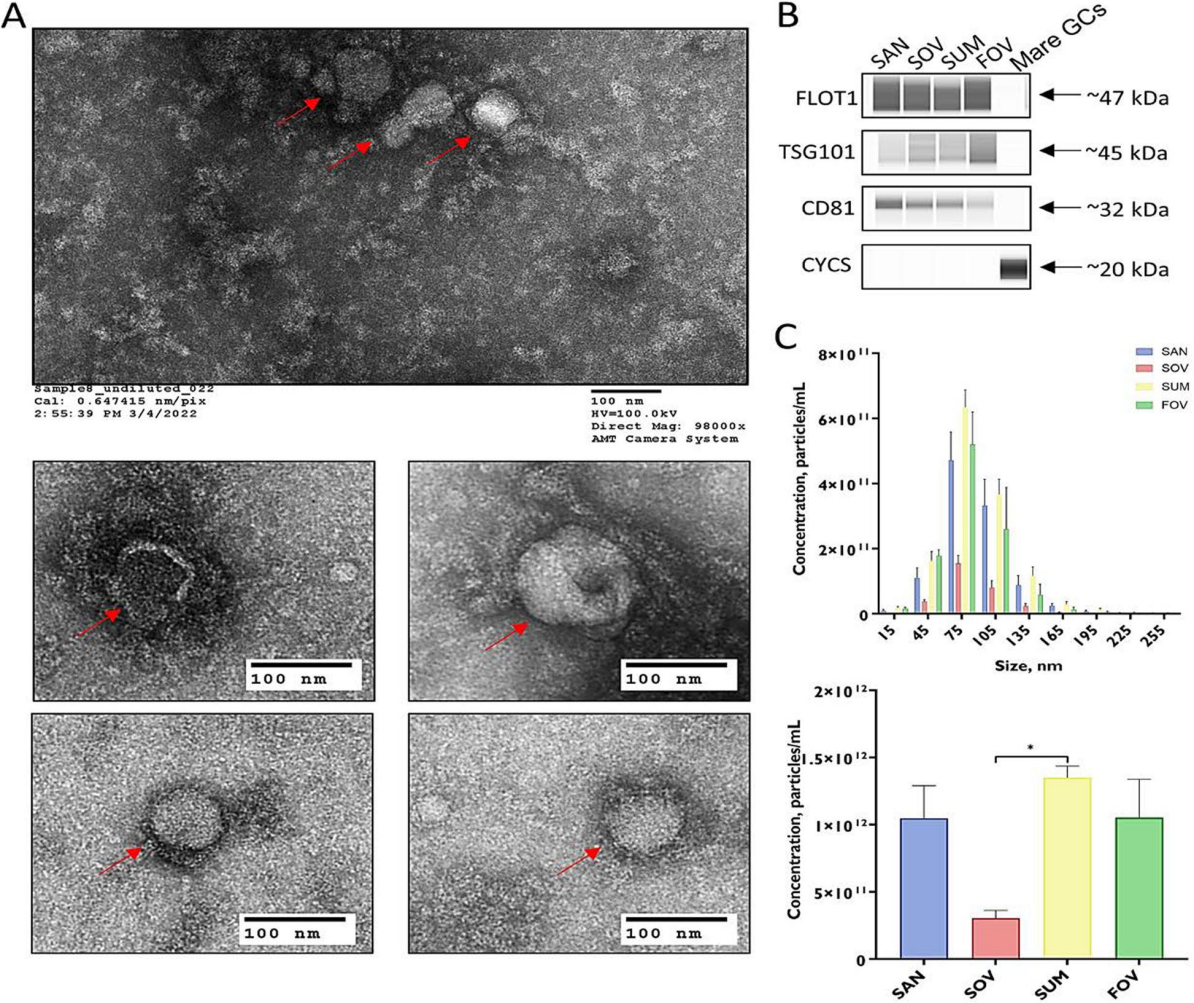
EV characterization. **A** Transmission electron microscope (TEM) imaging shows EVs with typical structure (red arrow), scale bar = 100 nm. **B** Western blot analysis of EVs marker proteins CD81, FLOT-1, and TSG101 and cellular protein contamination indicator CYCS. **C** The NTA analysis reveals the size distribution (upper panel) and concentration (lower panel) of the isolated EVs, with the asterisk indicating a significant difference between groups. Data were analyzed and graphs were generated using GraphPad Prism version 8.4.2 (GraphPad; San Diego, CA, USA). Statistical differences were assessed between the mean values of more than two groups using One-way Analysis of Variance (ANOVA) followed by Tukey’s Multiple Comparisons tests. Data are presented as the Mean ± SEM of biological replicates. Statistical significance was identified at *P* ≤ 0.05

The FF-EVs isolated from mares in SAN, SOV, SUM, and FOV underwent thorough morphological and molecular characterization, strictly following the guidelines of the International Society of Extracellular Vesicles (ISEV) [47]. A detailed summary of the FF-EVs’ characterization is presented in Fig. 1, highlighting the use of TEM (A), immunoblotting (B), and NTA (C) techniques for all samples. The isolated FF-EVs exhibited nanoscale, spherical structures with high purity and expressed typical exosomal protein markers, including CD81, FLOT-1, and TSG101, while showing the absence of the mitochondrial cellular contamination marker CYCS. Each seasonal group displayed varying EV sizes, averaging 85.9 nm for SAN, 81.2 nm for SOV, 84.4 nm for SUM, and 79.6 nm for FOV. The SOV-derived samples had the lowest EV concentration, averaging 3.03 × 10^11^ particles/mL, which significantly differed from the EV concentration in the SUM samples, averaging 1.35 × 10^12^ particles/mL (*P* < 0.05). Nevertheless, the EV concentration in the SOV samples did not significantly differ from that of the SAN and FOV groups.

### Sequencing and miRNA expression profiles

**Fig. 2:**
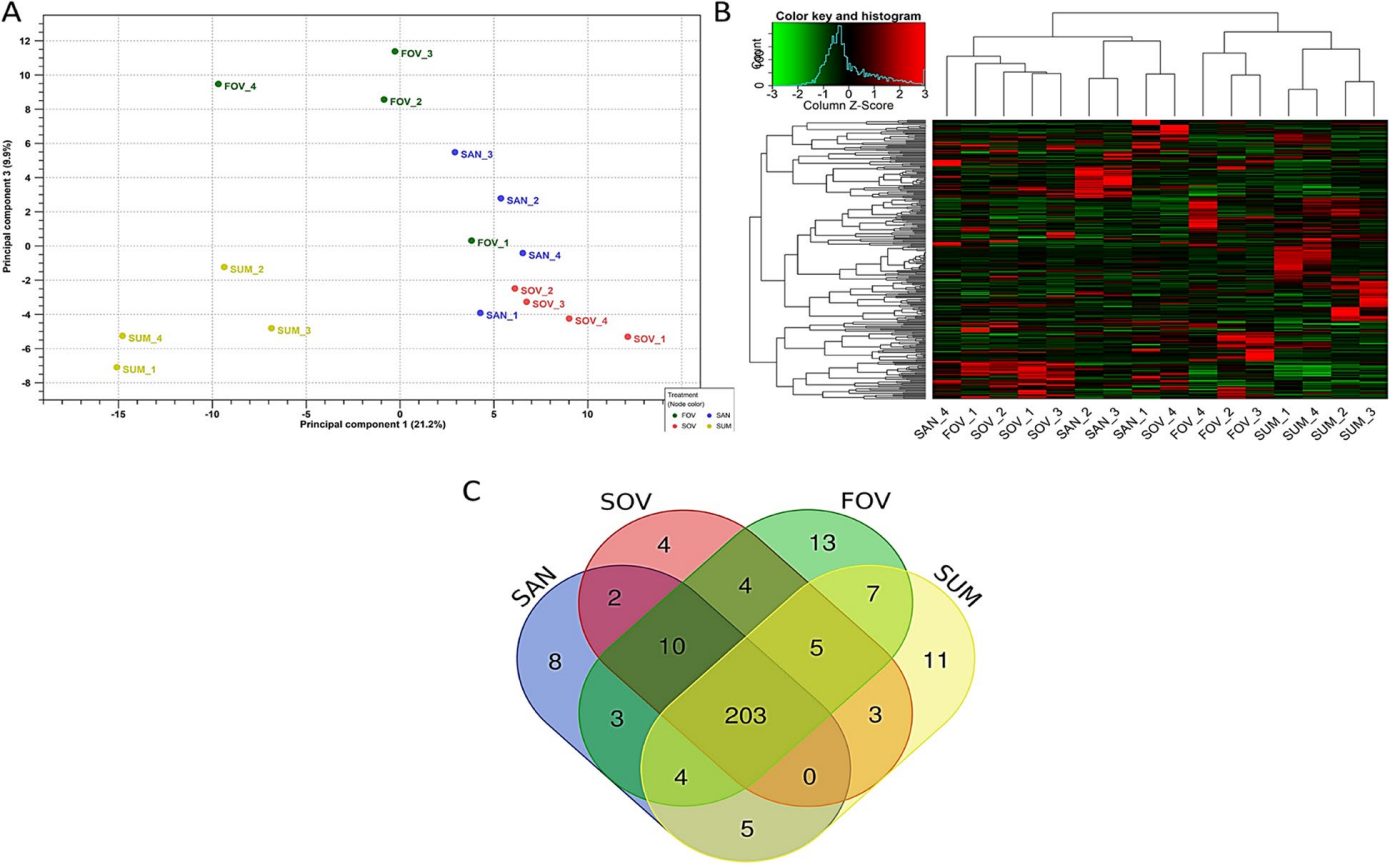
Principal component analysis (PCA), heatmap, and miRNA expression profiles. Small-RNA sequence data and miRNA expression profile overviews. **A** Principal component analysis. **B** Heatmap and hierarchical clustering of expressed miRNAs. Red and green colors represent high and low expressed miRNAs, respectively. **C** miRNA expression profiles in each seasonal group. Experimental groups are represented as SAN (Spring Anovulatory), SOV (Spring Ovulatory), SUM (Summer), and FOV (Fall Ovulatory) seasons. EVs and miRNAs were obtained from four independent samples, harboring different suffices (1, 2, 3, or 4) in each group. In the FOV group, however, FOV_1 was removed from further analyses (Fig. 2B and 2C) due to similarity to the SAN group (PCA analysis)

The small RNA sequencing analysis revealed an average of 23 million raw reads, resulting in nearly 22 million clean reads after QC trimming per sample. On average, 70% of the reads are successfully mapped to the equine reference genome. In Fig. 2, the principal component analysis (Fig. 2A) and heatmap (Fig. 2B) display distinct miRNA expression profiles and sample clustering for each seasonal group, excluding the FOV_1 sample from further analysis. A total of 282 unique miRNAs were expressed (> 10 CPM), with 235 in the SAN, 231 in the SOV, 238 miRNAs in the SUM, and 249 in the FOV group (Fig. 2C). Around 72% (203) of total detected miRNAs were found in all groups, and 13 of the 20 most abundant miRNAs were common to all datasets (Table 1). Among these, six miRNAs (miR-148a, miR-143, miR-99a, miR-192, miR-21, and miR-10b) accounted for 61%–72% of all 20 miRNA expressions. MiR-143 had the lowest expression level in the SUM group (3.8%) compared to 32.1% in the other groups, while miR-192 showed a similar pattern with 7.2% in the SAN group versus 31% in the others (Table 1). A detailed list of all expressed miRNAs per season is provided in Additional file 2.

**Table 1:** List of top 20 most abundant FF-EV miRNAs from different seasonal groups

**Name**	**SAN**	**Name**	**SOV**	**Name**	**SUM**	**Name**	**FOV**
**eca-miR-148a**	153217.5	**eca-miR-143**	220236.4	**eca-miR-148a**	185229.96	**eca-miR-148a**	130802.1
**eca-miR-99a**	152297.6	**eca-miR-99a**	139358.2	**eca-miR-21**	110374.93	**eca-miR-143**	124999.3
**eca-miR-143**	148564.7	**eca-miR-148a**	127656.7	**eca-miR-99a**	88377.861	**eca-miR-99a**	85233.95
**eca-miR-21**	66253.57	**eca-miR-21**	112097.5	**eca-miR-192**	61590.064	**eca-miR-192**	59664.31
**eca-miR-10b**	32721.78	**eca-miR-26a**	41150.8	**eca-miR-10b**	59013.673	**eca-miR-21**	47142.89
eca-let-7c	32308.23	**eca-miR-10b**	31779.63	**eca-miR-423-5p**	47375.737	eca-miR-100	33616.31
**eca-miR-26a**	31335.63	**eca-let-7 g**	27746.73	**eca-miR-10a**	38047.633	**eca-miR-10b**	27615.29
**eca-miR-10a**	27625.71	**eca-miR-423-5p**	25721.44	eca-miR-486-5p	28771.487	**eca-miR-423-5p**	27610.03
**eca-let-7 g**	23924.93	**eca-let-7f**	23289.82	eca-miR-218	27141.321	**eca-miR-10a**	24416.9
**eca-miR-423-5p**	20638.59	**eca-miR-27b**	23034.01	eca-miR-215	22536.951	**eca-miR-26a**	23407.8
**eca-let-7a**	18658.87	**eca-miR-192**	19979.72	**eca-let-7 g**	21758.141	eca-miR-215	22890.12
eca-miR-100	17932.95	**eca-let-7a**	18330.15	eca-miR-128	21423.475	eca-miR-24	18177.65
**eca-let-7f**	16062.56	eca-let-7c	16168.63	**eca-miR-26a**	19879.521	**eca-let-7 g**	17308.86
**eca-miR-27b**	14922.12	eca-miR-100	14713.95	**eca-miR-143**	19513.78	**eca-miR-27b**	16276.16
eca-miR-9a	14392.11	**eca-miR-10a**	13075.2	**eca-miR-27b**	19296.781	eca-miR-128	14358.85
eca-miR-1	13990.02	eca-miR-215	12377.61	**eca-miR-7f**	18443.203	eca-miR-27a	12482.74
**eca-miR-192**	10933.04	eca-miR-1	9928.538	eca-miR-22	18431.73	eca-miR-451	12127.98
eca-miR-30d	10138.15	eca-miR-126-3p	9608.478	eca-miR-24	17516.918	**eca-let-7f**	12126.65
eca-miR-24	9319.776	eca-miR-199a-3p	9071.105	eca-let-7c	14230.575	**eca-let-7a**	11556.09
eca-miR-128	7818.761	eca-miR-30d	8234.176	**eca-let-7a**	12710.04	eca-miR-486-5p	10986.71

The 20 most abundant miRNAs are listed from the highly to the lesser abundant in each season, measured as Count Per Million (CPM). The 13 commonly shared miRNAs are bolded. Experimental groups are represented as SAN (Spring Anovulatory), SOV (Spring Ovulatory), SUM (Summer), and FOV (Fall Ovulatory) seasons

### Differential expression, target gene prediction, and bioinformatic analyses

**Fig. 3:**
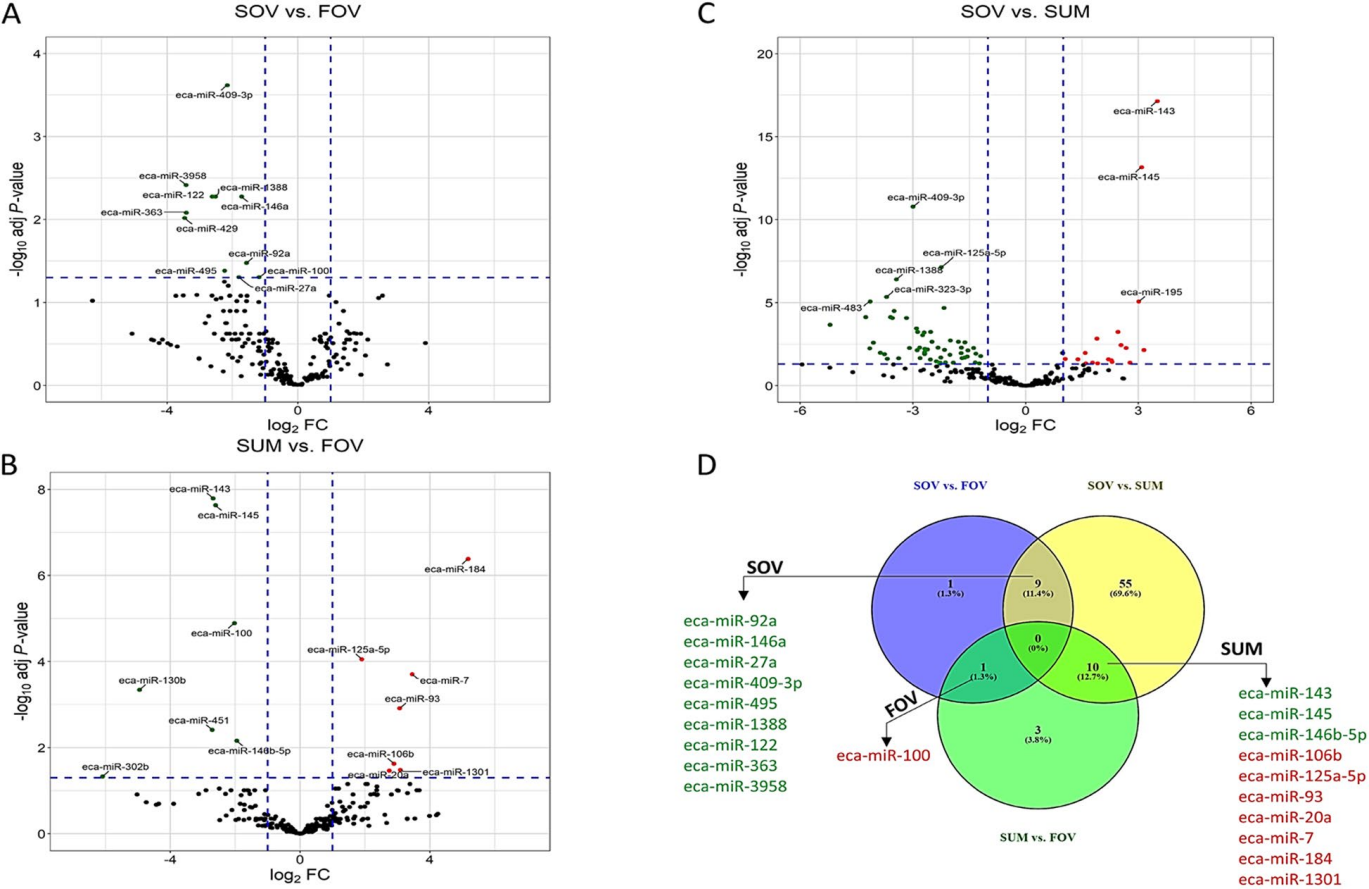
Differential expression and group comparisons. Volcano representations of pairwise comparisons between SOV and FOV (**A**), SUM and FOV (**B**), and SOV and SUM (**C**) are shown. The Venn diagram (**D**) provides the lists of miRNAs that are shared between pairwise comparisons (Red = downregulation and Green = upregulation). Experimental groups are represented as SAN (Spring Anovulatory), SOV (Spring Ovulatory), SUM (Summer), and FOV (Fall Ovulatory) seasons

**Fig. 4:**
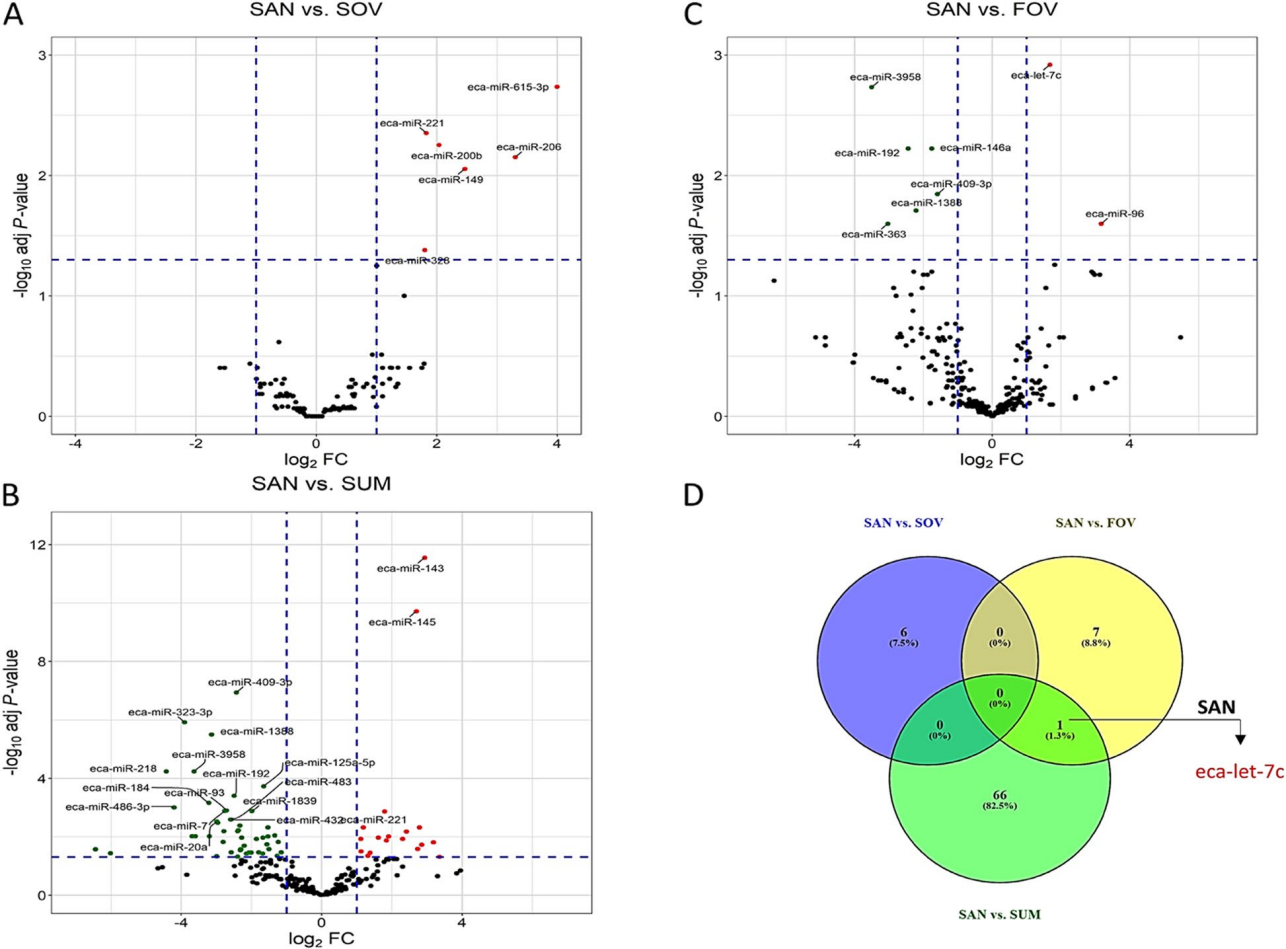
Differential expression and group comparisons. Volcano representations of pairwise comparisons between SAN and SOV (**A**), SAN and SUM (**B**), and SAN and FOV (**C**) are shown. The Venn diagram (**D**) shows the miRNAs that are shared between pairwise comparisons (Red = downregulation and Green = upregulation). Experimental groups are represented as SAN (Spring Anovulatory), SOV (Spring Ovulatory), SUM (Summer), and FOV (Fall Ovulatory) seasons

**Fig. 5:**
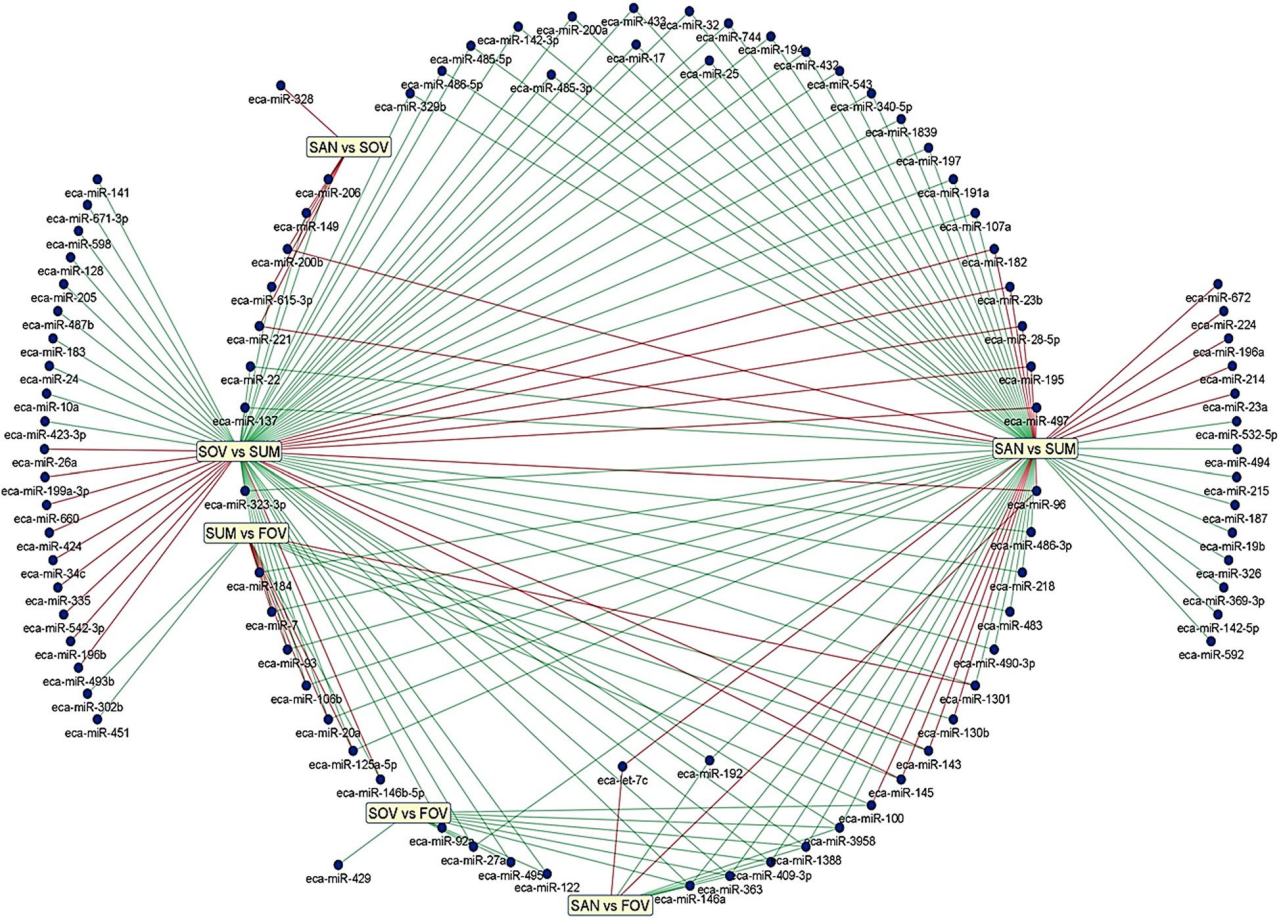
All differentially expressed (DE) miRNAs. All DE-miRNAs from the six different comparisons. Up- and down-regulated miRNAs from each comparison are presented as red and green lines, respectively. Experimental groups are represented as SAN (Spring Anovulatory), SOV (Spring Ovulatory), SUM (Summer), and FOV (Fall Ovulatory) seasons

The volcano plots in Figs. 3 and 4 depict the results of pairwise comparisons. A total of 97 unique miRNAs were found to be differentially expressed (FC > 2.0, FDR < 0.05, CPM > 10), with 11, 14, 74, 06, 67, and 8 miRNAs identified in SOV vs. FOV (Fig. 3A), SUM vs. FOV (Fig. 3B), SOV vs. SUM (Fig. 3C), SAN vs. SOV (Fig. 4A), SAN vs. SUM (Fig. 4B), and SAN vs. FOV (Fig. 4C), respectively. Venn diagrams in Figs. 3D and 4D illustrate the list of differentially expressed miRNAs shared between two comparisons. These miRNAs were either downregulated (in green) or upregulated (in red) in the joint seasonal group of both comparisons. Additionally, all differentially expressed miRNAs in the six comparisons are presented in Fig. 5 and Additional file 3. Among the findings, miR-143 and miR-192 were the two most abundant dysregulated in pairwise comparisons. For example, miR-143 was significantly downregulated in the SUM group compared to SOV (−11.33 FC, FDR = 7.3 × 10^−18^), SAN (−7.64 FC, FDR = 2.78 × 10^−12^), and FOV (−6.43 FC, FDR = 1.62 × 10^−8^). Similarly, miR-192 expression was downregulated in the SAN group compared to SUM (−5.63 FC, FDR = 3.9 × 10^−4^) and FOV (−5.46 FC, FDR = 5.97 × 10^−3^), as well as in the SOV group compared to SUM (−3.08 FC, FDR = 2.02 × 10^−2^). Additionally, several miRNAs appeared unique (e.g., miR-328 and miR-451) or paired (e.g., 302b and miR-429) in different comparisons, such as SAN vs. SOV, SUM vs. FOV, and SOV vs. FOV.

The subsets of differentially expressed miRNAs, including miR-143 and miR-192 (among the top 20 most abundant), as well as miR-149, miR-200b, miR-206, miR-221, miR-328, and miR-615 (spring transition), were chosen for target gene prediction and bioinformatic analyses. Results in Table 2, miR-143, targeting 74 genes, are mainly associated with post-translation modifications like phosphoprotein, isopeptide bound, Ubl conjugation, and methylation. Conversely, miR-192, targeting 49 genes, did not show significant enrichments. All six miRNAs upregulated in SAN vs. SOV targeted 634 genes, significantly enriched in diverse biological functions including DNA-, RNA-, and miRNA- “binding”, “transcription factor activity”, “sequence-specific DNA binding”, and post-translation modifications such as acetylation and methylation. The specific upregulation of miR-328 in SAN (vs. SOV; +3.48 FC), targeting 46 genes, showed significant enrichment in the “endoplasmic reticulum” cellular components. Additionally, the specific upregulation of miR-302b in SUM (vs. FOV; −68.13 FC) targeted 118 genes, enriched in various biological pathways related to cellular component (i.e., “nucleoplasm”), molecular function (i.e., “DNA-binding,” “transcription factor activity,” and “sequence-specific DNA binding”), and biological process (i.e., transcription and transcription regulation). Both miR-100 and let-7c were significantly dysregulated in multiple comparisons. MiR-100 was downregulated in SOV vs. FOV (−2.28 FC) and SUM vs. FOV (−4.06 FC) and upregulated in SAN vs. SUM (+2.17 FC), targeting 20 genes significantly enriched for “protein binding”. Meanwhile, let-7c was upregulated in SAN vs. FOV (+3.2 FC) and vs. SUM (+2.27 FC), targeting 311 genes enriched in various biological pathways, including the “p53 signaling pathway”, “glutamatergic synapse”, “response to gamma radiation”, and “endoplasmic reticulum.”

**Table 2:** Enrichment analysis of selected miRNA clusters

**miRNA**			**Seasonal comparisons**	**Biological pathways (*P* < 0.1)**
**Expression status**		**IDs** (# of Gene targets)	(Expression in 1^st^ stated group)	
Abundant	DE	miR-143 (74)	SUM-SOV (Down) SUM-SAN (Down) SUM-FOV (Down)	➢ KW-1017 ~ Isopeptide bond (PTM) ➢ KW-0832 ~ Ubl conjugation (PTM) ➢ KW-0597 ~ Phosphoprotein (PTM) ➢ KW-0488 ~ Methylation (PTM)
		miR-192 (49)	SAN-FOV (Down) SOV-SUM (Down) SAN-SUM (Down)	No significant enrichments
Transitional	Spring (DE)	miR-149 miR-200b miR-206 miR-221 miR-328 miR-615 (Total of 634)	SAN-SOV (Up)	➢ KW-0832 ~ Ubl conjugation (PTM) ➢ KW-1017 ~ Isopeptide bond (PTM) ➢ GO:0000381 ~ Regulation of alternative mRNA splicing (BP) ➢ KW-0694 ~ RNA-binding (MF) ➢ KW-0227 ~ DNA damage (BP) ➢ KW-0238 ~ DNA-binding (MF) ➢ GO:0000785 ~ chromatin (CC) ➢ GO:0003700 ~ transcription factor activity, sequence-specific DNA binding (MF) ➢ GO:0008139 ~ nuclear localization sequence binding (MF) ➢ GO:0035198 ~ miRNA binding (MF)
				➢ KW-0597 ~ Phosphoprotein (PTM) ➢ KW-0007 ~ Acetylation (PTM) ➢ KW-0488 ~ Methylation (PTM)
	Spring (Specific)	miR-328 (46)	SAN-SOV	➢ KW-0256 ~ Endoplasmic reticulum (CC)
	Fall (Specific)	miR-451 (1)	SUM-FOV	➢ Interleukin 6 receptor (IL6R)
		miR-302b (118)	SUM-FOV	➢ KW-0010 ~ Activator (MF) ➢ KW-0805 ~ Transcription regulation (BP) ➢ KW-0804 ~ Transcription (BP) ➢ KW-0597 ~ Phosphoprotein (PTM) ➢ GO:0003700 ~ transcription factor activity, sequence-specific DNA binding (MF) ➢ GO:0003677 ~ DNA binding (MF) ➢ GO:0005654 ~ Nucleoplasm (CC) ➢ GO:0005634 ~ Nucleus (CC) ➢ GO:0000978 ~ RNA polymerase II core promoter proximal region sequence-specific DNA binding (MF) ➢ GO:0003682 ~ chromatin binding (MF) ➢ GO:0001228 ~ transcriptional activator activity, RNA polymerase II transcription regulatory region sequence-specific binding (MF)
		miR-429 (none)	SOV-FOV	N/A
Multi-seasonal		Let-7c (311)	SAN-FOV SAN-SUM	➢ GO:0098978 ~ glutamatergic synapse (CC) ➢ GO:0005783 ~ endoplasmic reticulum (CC) ➢ GO:0010332 ~ response to gamma radiation (BP) ➢ KW-0597 ~ Phosphoprotein (PTM) ➢ hsa04115: p53 signaling pathway (KEGG) ➢ GO:0005739 ~ mitochondrion (CC) ➢ GO:0005829 ~ cytosol (CC) ➢ GO:0005654 ~ nucleoplasm (CC) ➢ GO:0005737 ~ cytoplasm (CC)
		miR-100 (20)	SOV-FOV SUM-FOV SAN-SUM	➢ GO:0005515 ~ protein binding (MF)

*DE* Differentially expressed or Differential expression, *Down* Downregulated, *Up* Upregulated, *CC* Cellular component, *MF* Molecular function, *BP* Biological process, *PTM* Post-translational modification, *GO* Gene Ontology, *KW* Key word, *KEGG* Kyoto Encyclopedia of Genes and Genomes. Experimental groups are represented as *SAN* Spring Anovulatory, *SOV* Spring Ovulatory, *SUM* Summer, and *FOV* Fall Ovulatory seasons

**Fig. 6:**
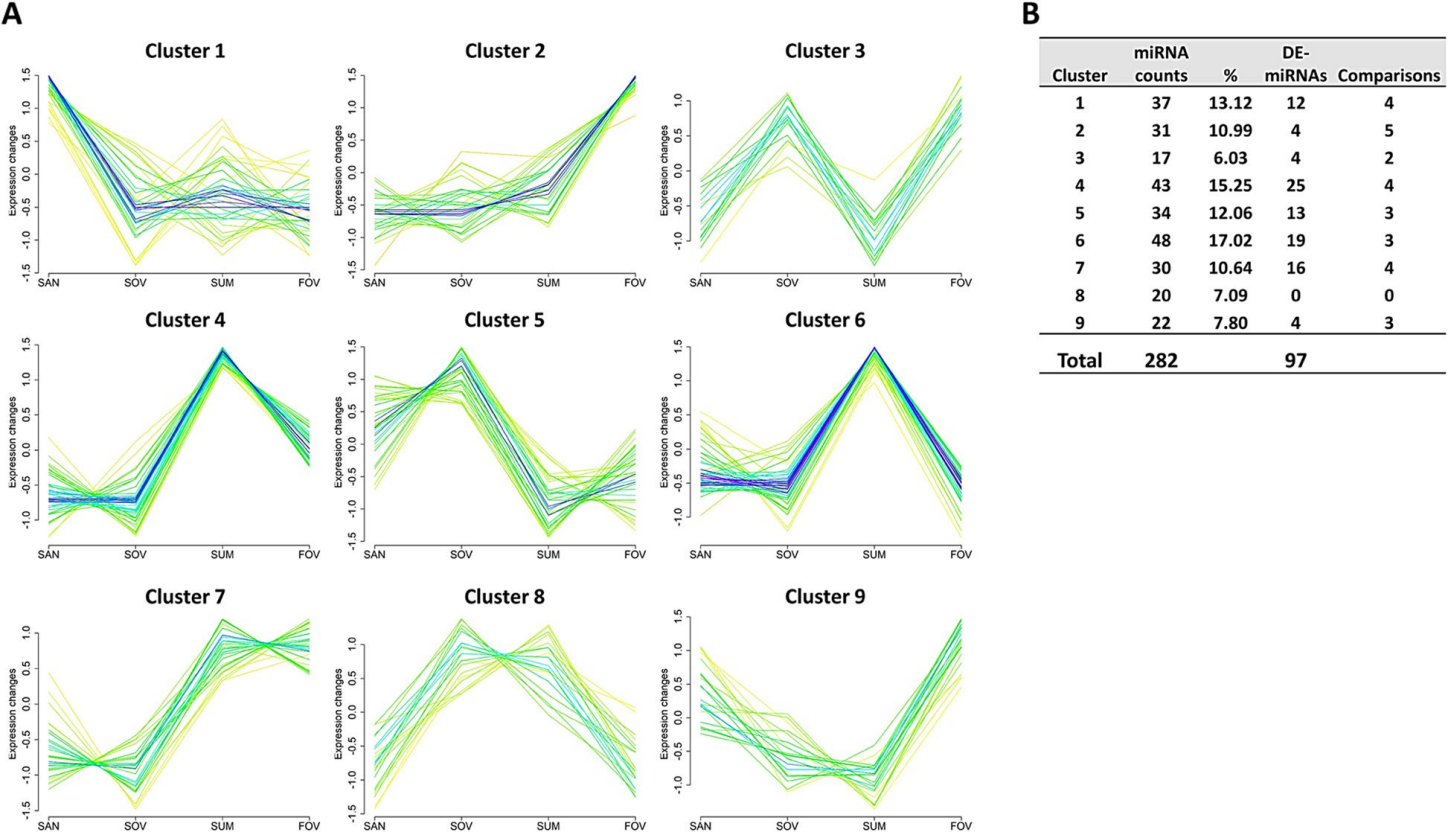
Clustering analysis of extracellular vesicle–coupled microRNAs across the different seasons. **A** Nine different miRNA expression patterns in the follicular fluid extracellular vesicles across the seasons. **B** The counts and percentages of expressed microRNAs (miRNAs) and the differentialy expressed (DE) miRNAs from the different comparisons in each cluster. Each line represents one miRNA that fits (better: red, purple, and blue lines; lesser: yellow or green lines) with its pattern of expression within the cluster. Experimental groups are represented as SAN (Spring Anovulatory), SOV (Spring Ovulatory), SUM (Summer), and FOV (Fall Ovulatory) seasons

**Fig. 7:**
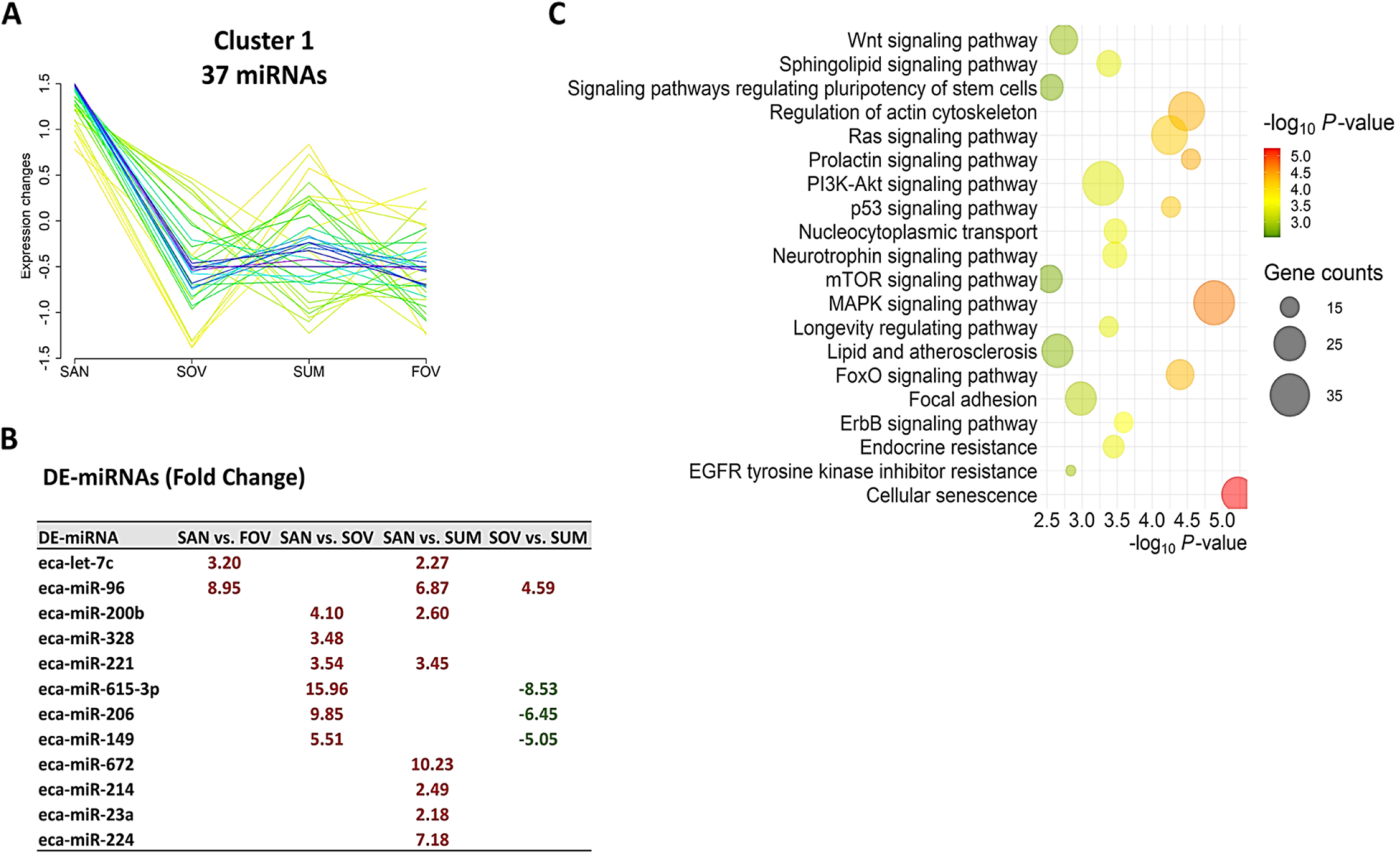
Cluster 1 of microRNAs (miRNAs). A group of 37 miRNAs exhibited a sharp reduction in their expression from the SAN till the FOV seasons (**A**). Among this cluster, 12 miRNAs were differentially expressed (DE) in four comparisons (**B**). Top 20 pathways associated with the DE-miRNAs target genes of this cluster (**C**). Experimental groups are represented as SAN (Spring Anovulatory), SOV (Spring Ovulatory), SUM (Summer), and FOV (Fall Ovulatory) seasons

**Fig. 8:**
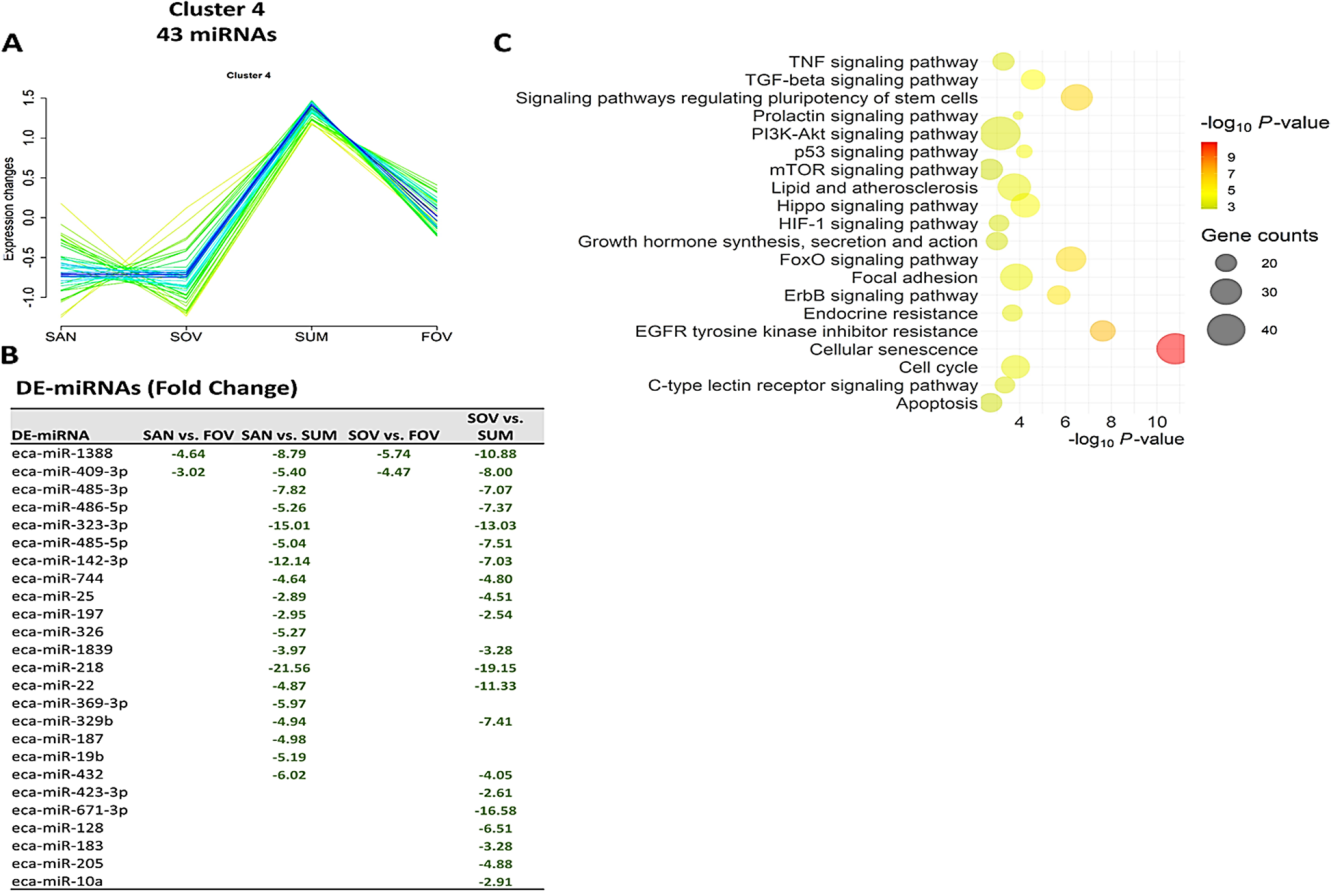
Cluster 4 of microRNAs (miRNAs). A group of 43 miRNAs exhibited a sharp increase in their expression during the summer and then a slight reduction during the fall ovulatory season (**A**). Among this cluster, 25 miRNAs were differentially expressed (DE) in four comparisons (**B**). Top 20 pathways associated with the DE-miRNAs target genes of this cluster (**C**). Experimental groups are represented as SAN (Spring Anovulatory), SOV (Spring Ovulatory), SUM (Summer), and FOV (Fall Ovulatory) seasons

**Fig. 9:**
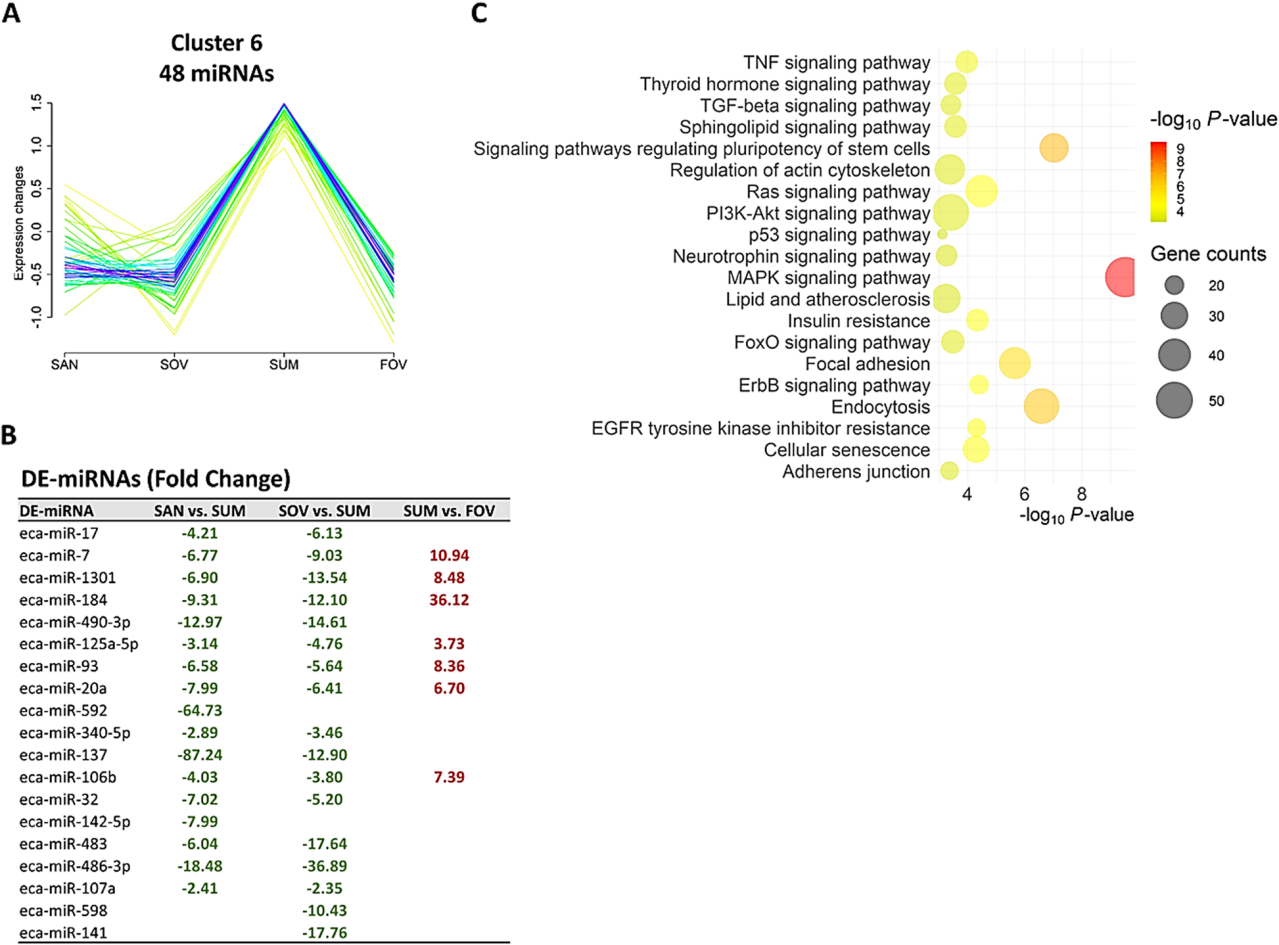
Cluster 6 of microRNAs (miRNAs). A group of 48 miRNAs exhibited a sharp increase in their expression during the summer compared to the other seasons (**A**). Among this cluster, 19 miRNAs were differentially expressed (DE) in three different comparisons (**B**). Top 20 pathways associated with the DE-miRNAs target genes of this cluster (**C**). Experimental groups are represented as SAN (Spring Anovulatory), SOV (Spring Ovulatory), SUM (Summer), and FOV (Fall Ovulatory) seasons

Cluster analysis revealed nine distinct patterns of miRNA expression across seasons (Fig. 6A), with a substantial number of miRNAs associated with each cluster (Fig. 6B). Three clusters demonstrating critical seasonal patterns displayed significant numbers of DE-miRNA (12, 25, and 19 for Clusters 1, 4, and 6, respectively), thereby allowing for further pathway analyses. Cluster 1 exhibited the highest expression levels of 37 miRNAs in the SAN group (Fig. 7A). In contrast, both Cluster 4 (Fig. 8A) and Cluster 6 (Fig. 9A) displayed similar highest expressions of 43 and 48 miRNAs, respectively, in the SUM group. DE-miRNAs were found in 4 (Clusters 1 and 4; Figs. 7B and 8B) and 3 (Cluster 6; Fig. 9B) different comparisons, leading to significant enrichments of various pathways (Figs. 7C, 8C, and 9C).

## Discussion

The molecular mechanisms governing ovarian dynamics during different seasons remain mysterious. Extracellular vesicles in follicular fluid are crucial in causing functional adjustments in target cells [48, 49]. A previous study demonstrated that mares’ follicular fluid-derived extracellular vesicles’ miRNA profiles are closely associated with follicle development, selection, and ovulation. However, little is known about EVs and their effectual miRNA profiles during the ovulatory and anovulatory seasons. This study compares miRNA cargo of extracellular vesicles collected from mares’ follicular fluid during ovulatory and anovulatory seasons, and significant seasonal variations are unveiled. These findings enhance our understanding of extracellular vesicle–coupled miRNA control over ovarian activity during seasonal reproductive changes.

### EV identification, total and abundant miRNA in equine follicular fluid across seasons

The follicular fluid contains vital components for oocyte and follicular maturation and cellular communication. Studies have shown that FF-EV–derived miRNAs are crucial in mediating cellular communication [50] and responding to stress within the follicle [51–53]. Our findings revealed a higher accumulation of EVs in the SUM group, which may implicate an increased EV release from the follicular cells in response to high environmental temperature. Previous in vivo and in vitro studies have demonstrated these EVs can shuttle protective signals in the form of miRNAs, which can induce tolerance to high temperatures if delivered to recipient cells [30, 31, 39, 54], indicating their potential to regulate the response to elevated thermal temperatures for maintaining intrafollicular homeostasis.

Our analysis found that 203 out of 282 (72%) of the uniquely detected miRNAs were shared across seasons, indicating that they may represent the core miRNA profile of equine FF-EVs, which may have a housekeeping role in follicular dynamics in mares irrespective of the season. During the winter-to-summer comparison, a similar core miRNA profile was observed in bovine FF-EV–derived miRNAs (89.6%: 232/259) [31]. Studies on equine [25] and porcine [55] follicle development also reported high percentages of shared miRNA across follicle developmental stages (82% and 86%, respectively). The expression levels of the number of these FF-EV–coupled miRNAs were altered across seasons (Cluster analysis), which observation was in agreement with previous reports in short-day breeder sheep [56, 57] and buffalo oocyte- and follicular cell-derived miRNAs [58]. Considering their involvement in various cancers, miRNAs have been suggested as potent regulators of follicular atresia and degeneration [59], playing a crucial role in follicular development, meiotic resumption, and ovulation by targeting genes involved in signaling pathways such as *AKT*, *WNT1*, *GDF9*, *BMP15*, *MAPK*, *ErbB*, *PTEN*, *PI3K-Akt*, *P53*, *mTOR*, *FOXO1*, and *TGFβ1* [60–62].

The current study found that miR-148a, miR-143, miR-192, miR-99a, miR-21, miR-10a, and miR-10b are abundant miRNAs across all seasons. These miRNAs regulate ovarian development, including ovarian cell proliferation and steroidogenesis [63–65]. They have been shown to have diverse beneficial effects on livestock species, such as sheep [66, 67] and pigs [68, 69]**,** with reported effects on embryo quality [28]. High expression levels of these miRNAs were found in FF-EVs of large antral follicles, granulosa, and luteal cells throughout folliculogenesis, and their upregulation in late corpus luteum and atretic follicles during SAN, SUM, and FOV was observed in various livestock species, including sheep [70], cows [31, 71], horses [25], and pigs [33]. Previous studies have indicated positive correlations between these miRNAs and oocyte maturation [72] and IVF outcomes [28], highlighting the potential of these abundant miRNAs as essential molecules in regulating ovarian function throughout all seasons and as potential markers for improving female fertility.

### Seasonal variations in FF-EV–coupled miRNAs

The cluster analysis found that about 34% of all detected miRNAs are differentially expressed across seasons, likely impacting follicular function and ovulatory capacity in various seasons. The discussion will focus on selected miRNAs with high abundance and significant differential expression, especially those related to spring (SAN vs. SOV) and fall (SUM vs. FOV) transitions or involved in selected clusters (1, 4, and 6) or comparisons across multiple seasons.

Two abundant miRNAs, miR-192 and miR-143, varied significantly across seasons. Despite no significant pathway enrichment of the miR-192 gene targets, its higher expression during SUM (vs. SAN and SOV) and FOV (vs. SAN) suggests a link to ovulation. Noteworthy, the miR-192 has been proposed as a potential biomarker for early pregnancy screening in sows due to its higher detection in blood serum [73]. On the other hand, the downregulation of miR-143 in SUM (vs. SAN, SOV, and FOV) is associated with post-translational modifications impacting protein functions, especially apoptosis. Overexpression of miR-143-3p has been found to have steroidogenesis inhibitory and pro-apoptotic effects, targeting intracellular signaling factors like *TGF-β* in porcine granulosa cells and follicular atresia [74], as well as *BMPR1A/SMAD1/5/8* in polycystic ovary syndrome (PCOS). This endocrine disorder leads to abnormal follicular development, chronic anovulation, and infertility [75]. Higher levels of miR-143-3p expression have also been reported in FF-EVs of women with PCOS [76]. Therefore, the contrasting expressions of miR-192 (upregulation) and miR-143 (downregulation) in healthy equine preovulatory follicles during summer could potentially serve as predictive markers for successful ovulation and promise improved early pregnancy screening methods in mammalian reproduction.

#### Spring transition

In this study, the principal component analysis (PCA) comparing the spring anovulatory season (SAN) and the spring ovulatory season (SOV) showed minimal overlap, regardless of the PC1/PC2 (Additional file 4) and PC1/PC3 combination. The factors contributing to the onset of normal ovarian cyclicity during the spring transition remain unknown. Here, we found that during this transition, specific miRNAs (miR-149, miR-200b, miR-206, miR-221, miR-328, and miR-615) were overexpressed in the SAN compared to the SOV season, suggesting their role in maintaining oocyte meiotic arrest and regulating the cell cycle. These miRNAs are identified in Cluster 1, characterizing the expression profiles of the anovulatory or non-breeding season. Twelve related differentially expressed miRNAs may promote cellular senescence over apoptosis by influencing pathways that regulate stable cell cycle arrest and/or cell survival. The overexpression of these miRNAs may contribute to maintaining oocytes in a meiotic arrest state (e.g., miR-206) through ERα targeting [77, 78], regulating the cell cycle by promoting proliferation or inducing arrest (e.g., miR-221, miR-149, and miR-615) via targets such as *FoxO*, *MAPK*, and estrogen signaling pathways [61], and ensuring the developmental transition from the follicular to luteal phase (e.g., miR-221-5p) through targeting genes associated with *Ras* and *PI3K-Akt* signaling pathways [79]. These differentially expressed miRNAs significantly enrich these signaling pathways during the spring transition and have been identified in the preovulatory follicle cells and FF-EVs of humans [80, 81], pigs [68, 82], and sheep [83, 84], playing a crucial role in regulatory processes. In the current study, these miRNAs exert their impacts through regulating target genes affecting biological functions such as post-translational modifications, RNA- and DNA-binding, DNA damage, chromatin, transcription activity, nuclear localization sequence binding, and miRNA binding, all associated with steroidogenesis and/or cell cycle [33, 81, 83]. These intricate regulatory roles are key to their function during follicular development and oocyte maturation [33, 85, 86], with the potential to positively influence female reproductive performance [85, 87, 88]. Nutritional studies have reported dysregulations of miR-328 and miR-200 impacting fertility outcomes [28], and miR-200 inspires further research through appropriate nutritional measures during the non-breeding season, as reported in sheep [83, 89].

#### Summer ovulatory season, the peak of breeding

Our research has identified 2 key Clusters, 4 and 6, shedding light on the distinct roles of miRNAs during the summer ovulatory season (SUM). The biological functions within both clusters converge on sustaining cell proliferation, growth, and survival processes in ovarian follicles. Cluster 4 is linked to cellular senescence, while Cluster 6 is linked to the MAPK signaling pathway and the regulation of pluripotency in stem cells, especially within the oocyte. These findings underscore the intricate balance between non-dividing oocytes and dividing somatic cells in the ovarian follicle during the peak of breeding. Understanding these clusters could enhance the sustainability of cell proliferation, growth, and survival processes in the follicles, potentially positively influencing seasonal fertility.

#### Fall transition

Preovulatory follicles in various species, including humans [90, 91], horses [20], cows [60], pigs [82], chickens [92], and mice [93, 94], have been found to contain specific miRNAs such as miR-302b, 429, and 451. The expression of miR-302b was specific to the transition from summer to fall ovulation, showing higher levels in the summer compared to the fall. In chickens, reduced expression of miR-302b in primordial germ cells has been linked to decreased cell proliferation and increased apoptosis, potentially contributing to the decline in intrafollicular processes leading to the end of the ovulatory season [92]. On the other hand, miR-429 expression was detected in both the spring and fall ovulatory seasons and has been associated with luteinizing hormone synthesis in the pituitary gland [62, 94], likely linked to the transition from the follicular phase to the luteal phase. Similarly, the overexpression of miR-451 in the summer compared to the fall suggests potential benefits during ovulation. In cows, miR-451 is overexpressed in large healthy follicles (12–17 mm) [95], while in women with endometriosis, decreased miR-451 expression in FF and oocytes is associated with reduced fertility potential such as decreased number of oocytes, fertilization rate, and embryo quality compared to healthy women [93]. Altogether, these findings emphasize the importance of further exploring these miRNAs’ functional roles and practical applications in reproductive health, especially the overexpression of miR-302b and miR-451 in the SUM group (compared to FOV) during ovulation.

#### Comparing multiple seasons

It was found that miR-100 and let-7c are significant miRNAs that are present in various ovarian cell types, FF, and FF-EVs and play a role in promoting follicle development and regulating ovarian follicle pathways in different species, including human [96], horses [25], and cows [31, 60]. MiR-100 has multiple roles in cells and may affect oocyte reprogramming through its interaction with SMARCA5, a key regulator of global chromatin structure [97, 98]. Previous studies showed that miR-100 triggers the resumption of meiosis in human oocytes by negatively regulating genes encoding inhibitory factors of follicular maturation via *mTOR* [96, 99, 100] or supports tumor suppression in ovarian cancer via *mTOR*, inducing apoptosis [99]. Thus, controlling the *mTOR* by reduced expression of miR-100 during SOV and SUM could maintain the oocyte in meiosis arrest for further cytoplasmic maturation to optimal developmental competence acquisition and the survival of other follicular cells. On the other hand, the overexpression of let-7 family members (let-7c, let-7a-5p, and let-7 g-5p) in FF-EVs is directly linked to human oocyte failure to fertilize [28, 101], with significantly higher expressions in developmentally arrested oocytes at the GV stage, as compared to their MI and MII counterparts [102]. These reports support the findings of the present study, which identifies the upregulation of let-7c during anovulatory (SAN) versus ovulatory seasons (SUM and FOV) as potentially contributing to oocyte meiotic arrest during SAN.

## Conclusions

This study presents the miRNA profiles of equine FF-EVs collected during anovulatory or non-breeding (SAN) and ovulatory or breeding (SOV, SUM, and FOV) seasons. The EVs were isolated from dominant 30–34 mm follicle sizes, which are absent in ovaries during deep winter anestrus (contain only follicles < 20 mm in diameter). The study has unveiled seasonal dynamic patterns of FF-EV–coupled miRNAs, which are pivotal for advancing fertility research. A group of miRNAs appeared as potential regulators of spring transition (SAN-SOV), with specific miRNAs upregulated in the SAN season (miR-149, miR-200b, miR-206, miR-221, miR-328, and miR-615). Furthermore, the study has revealed that specific miRNAs play a crucial role in maintaining continued folliculogenesis, leading up to ovulation during the ovulatory seasons (SOV, SUM, and FOV), with consistent downregulation compared to SAN (miR-100, let-7c, and miR-143) and FOV (miR-302b) and upregulation compared to SAN (miR-192). These findings shed light on the potential involvement of EV-coupled miRNAs in seasonal variations on breeding and fertility, offering insights into ovulatory failure mechanisms.

## Supplementary Information

**Additional file 1.** Detailed materials and methods.

**Additional file 2.** Expressed miRNAs in each seasonal group represented as the average CPM value.

**Additional file 3.** Total differentially expressed miRNAs.

**Additional file 4.** Principal component analysis: P1/P2.

## Data Availability

The miRNA sequencing dataset generated during the current study, including the raw FASTQ files and processed CSV files, was deposited in the National Center for Biotechnology Information (NCBI) Gene Expression Omnibus (GEO) database (https://www.ncbi.nlm.nih.gov/geo/) for open-access using the accession number GSE249220. Experimental procedures related to EV experiments have been submitted to the EV-TRACK knowledgebase (https://evtrack.org/) (EVTRACK ID: EV231010). Additional data are included in Additional file 1 (Detailed materials and methods), Additional file 2 (Expressed miRNAs in each seasonal group represented as the average CPM value), Additional file 3 (Differentially expressed miRNAs in all comparisons), and Additional file 4 (Principal component analysis: P1/P2).

## Abbreviations

BMPR1A: Bone morphogenetic protein receptor type 1A
CPM: Count per million
DE: Differential expression
DNA: Deoxyribonucleic acid
EV: Extracellular vesicles
FF: Follicular fluid
FOV: Fall ovulatory
HRP: Horseradish peroxidase
LH: Luteinizing hormone
MI: Metaphase I
MII: Metaphase II
PCOS: Polycystic ovary syndrome
RNA: Ribonucleic acid
SAN: Spring anovulatory
SMARCA5: SWI/SNF-related matrix-associated actin-dependent regulator of chromatin A5
SOV: Spring ovulatory
SUM: Summer
TEM: Transmission electron microscope
TGF-b: Transforming growth factor-beta
Ubl: Ubiquitin-like
