# Supplementary material for: Seasonal influence on miRNA expression dynamics of extracellular vesicles in equine follicular fluid
Source: J Anim Sci Biotechnol. 2024 Oct 9;15:137. doi: 10.1186/s40104-024-01097-2 (PMC11462823; doi:10.1186/s40104-024-01097-2)
Supplement: Supplementary file 1 — Additional file 1. Detailed materials and methods. [file 40104_2024_1097_MOESM1_ESM.docx]

**Materials and methods**

**Animals**

Nineteen multiparous Quarter Horse mares weighing 400 to 600 kg, spanning the ages 8 to 14 years old (10.6 ± 1.8 years; mean ± SD), were housed on pasture in the northern hemisphere, under natural light conditions, with ad libitum access to fresh water and trace-mineralized salt. As determined by ultrasonographic examination, animals had normal reproductive tracts. All mares were used for in vivo collections of FF samples during the SAN, SOV, SUM, and FOV seasons.

**Transrectal ultrasonography and seasonal grouping**

Follicular fluids (FF) were collected in vivo from pre-ovulatory follicles with a diameter of 30–35 mm (*n* = 6–12/group). Collections were carried out in different seasons: SAN (*March*, the transition season before the spring ovulatory season), SOV (*April and May*, the beginning of the ovulatory season, with regular cyclic activity), SUM (*June to July*, the peak ovulatory season), and FOV (*September*, the final period of the ovulatory season), before the mares approached the receding phase (fall anovulatory). Ablations of ovarian follicles were performed during the non-breeding (SAN) and the breeding (SOV, SUM, and FOV) seasons, 10–11 days after the ovulation of the previous cycle, to induce a new follicular wave, as described previously [36]. Thereafter, daily ultrasonographic tracking was performed using a duplex B-mode and color-Doppler ultrasound machine (Aloka SSD-3500; Hitachi Aloka Medical America, Inc., Wallingford, CT, USA) equipped with a finger-mounted 3.5–10 MHz convex-array transducer (UST-995-7.5) until the dominant follicle reached the pre-ovulatory diameter size. Samples were collected only from animals showing continuous ultrasonographic follicle growth for at least three consecutive days, uterine edema (estrus-like), and the absence of a corpus luteum at the time of FF collection.

**Follicular-fluid sample collection**

All mares were adequately prepared and sedated before follicular ablation and FF sampling procedures, as described in previous studies [37, 38]. Briefly, antral follicles were measured and aspirated using a sterile 12-G needle mounted on a 5–10 MHz transvaginal ultrasound-guided convex array transducer (Aloka UST-987-7.5). Only the tip of the 12-G needle was inserted into the follicle's antrum once the targeted follicle appeared on the ultrasound screen, and the leaking FF was promptly collected into a sterile vial. Following the abovementioned restrictions, only 6 to 12 mares per seasonal group were used, resulting in 42 clear FF samples without visible blood contamination throughout the year were used. The collected FF samples were immediately centrifuged (1,600 × *g* for 10 min at 4 °C), and the supernatants were then subjected to additional centrifugation (3,200 × *g* for 15 min at 4 °C) and stored at −80 °C for subsequent analysis

**Isolation of follicular fluid-extracellular vesicles**

The experiment combined two to three follicular fluid (FF) samples of individual mares (0.5 mL) to create four biological replicates (1.0–1.5 mL/pool) for isolating extracellular vesicles (EVs). The FF samples underwent a series of centrifugation steps, starting at 500 × *g* for 5 min to eliminate cells, followed by centrifugation at 4,000 × *g* for 5 min to remove cellular debris. Subsequently, supernatant FFs were filtered through a 0.22-µm sterile filter to remove particles larger than 200 nm. The isolated EVs were obtained by subjecting the samples to centrifugation at 25,000 × *g* for 30 min at 4 °C. For EV isolation, a 2-mL portion of the pre-centrifuged FF samples underwent an ultracentrifugation process at 120,000 × *g* for 70 min at 4 °C using a Beckman SWTi55 rotor. The EV pellet was washed with sterile PBS and centrifuged at 120,000 × *g* for 70 min. Finally, the EVs were resuspended in 500 µL of PBS and stored at −80 °C until further characterization and analysis.

**Morphological and molecular characterization of FF-EVs**

EV samples were subjected to protein separation and immunodetection of EV-specific protein markers (CD81, FLOT-1, and TSG101) and the absence of a cell-specific marker protein, cytochrome C (CYCS) using the JESS Simple Western^™^ instrument (ProteinSimple^®^, Bio-Techne, Minneapolis, MN, USA). Briefly, a total of 10 µL of protein from each sample was extracted using RIPA lysis buffer (Sigma-Aldrich; St. Louis, MO, USA). Following the manufacturer's protocol, the instrument was operated under JESS’s Assay Module Protein Normalization (AM-PN001) using the 25 capillary cartridges (R&D Systems, United States: part number SM-PN01-1). Three μL of protein lysate was used for each sample per marker: Anti-CD81 rabbit polyclonal (1:75, System Biosciences, USA; Cat# EXOAB-CD81A-1), Anti-FLOT1 rabbit polyclonal (1:20, System Biosciences, USA; Cat# EXOAB-FLOT1-1), and Anti-TSG101 rabbit polyclonal (1:50, System Biosciences, USA; Cat# EXOAB-TSG101-1). The secondary Ab (anti-rabbit HRP) reagent was as per the kit’s instructions (ProteinSimple^®^, Bio-Techne, Minneapolis, MN, USA). The proteins were detected and analyzed using Compass for Simple Western software (USA).

Ten μL of each purified frozen-thawed EV samples was diluted in 9,990 μL of sterile PBS to measure their size and concentration, using the Zetaview Laser scattering microscope (Zetaview Particle Metrix, Germany) fitted with an LM14C laser. Each sample underwent 11 independent video measurements recorded at 11 different positions, with video files analyzed using the ZetaView software version 8.05.12.

The morphology and size of the frozen-thawed EVs were examined using a transmission electron microscope (TEM) following previously reported methods [39]. Briefly, a drop of 6–8 μL of purified EVs was placed on formvar/carbon-coated grids and allowed to stand for 1–2 min to absorb the EVs, followed by negative staining using 2% uranyl acetate. EVs on the carbon-coated grids were observed under an FEI/TFS Tecnai T12 Spirit TEM (FEI Company; Hillsboro, OR, USA), operating at 100 kV, with an AMT CCD. All relevant EV-related data from experiments using EVs from equine pre-ovulatory FF in various seasons have been submitted to the EV-TRACK knowledgebase (https://evtrack.org) under the EV-TRACK ID EV231010 [40].

**Total RNA extraction, library preparation, and sequencing**

EV samples from follicular fluid of animals in different seasons were subjected to total RNA isolation, including miRNAs, using a Norgen Exosomal RNA Isolation kit, per the manufacturer’s instructions (Norgen, Canada). Subsequently, on-column DNA digestion was conducted to eliminate genomic DNA contaminants. The RNA concentration and size distribution were analyzed using an Agilent RNA 6000 Pico kit in an Agilent 2100 Bioanalyzer (Agilent Technologies, Santa Clara, CA, USA). Following the manufacturer's instructions, small-RNA libraries were prepared for next-generation sequencing (NGS) using a TruSeq Small RNA Library Prep Kit (Illumina). The quantity and quality of the libraries were evaluated using a Qubit DNA HS Assay Kit in a Qubit 2.0 Fluorometer (Thermo Fisher Scientific) and an Agilent DNA High Sensitivity kit in an Agilent 2100 Bioanalyzer (Agilent Technologies), respectively. The precise concentration of the libraries was determined using quantitative PCR. Subsequently, the libraries were combined in equimolar ratios and sequenced in a single-end reads (50 bases) using a NovaSeq6000 sequencing instrument (Illumina, Inc., San Diego, CA, USA).

**Small RNAseq data analysis**

The FASTQ files were generated for each sample using the bcl2fastq software (Illumina Inc., San Diego, CA, USA), and subsequently checked for quality using FastQC tool version 0.11.9. Data analysis was performed using CLC Genomics Workbench, version 21. Raw sequencing reads were subjected to trimming based on quality score (Q‐score > 30), ambiguous nucleotides (maximum two nucleotides allowed), read length (≥ 15 nucleotides), and removal of adapter sequences. The reads were then mapped to the equine (*Equus caballus*) reference genome (EquCab3.0) and annotated against equine precursor and mature miRNAs listed in the mirBase database (release 22) using the CLC Genomics Workbench RNA-Seq Analysis and Quantify miRNA tools, with default software parameters applied. Raw expression data were normalized using the trimmed mean of M‐values normalization method (TMM normalization) [41] and presented as TMM-adjusted Counts Per Million (CPM). Differential expression analysis was performed using the CLC Genomics Workbench Differential Expression tool. MiRNAs with a fold change (FC) ≥ 2 and *P*‐adjusted value (FDR) < 0.05 [42] were considered differentially expressed (DE). The raw FASTQ and processed CSV files have been deposited in the NCBI’s Gene Expression Omnibus (GEO) under accession number GSE249220.

**Cluster analysis, target gene prediction, and ontological classification**

All identified miRNAs were grouped into distinct clusters based on their expression patterns across different seasons using the Mfuzz Bioconductor package [43]. The targeted genes of the differentially expressed miRNAs were determined by matching them with human homologous miRNAs in the miRWalk database [44]. Validated target genes from miRTarBase (version 7.0) and predicted target genes from TargetScan (version 7.1) and miRDB (release 5.0) were chosen from miRWalk for pathway analysis using the DAVID bioinformatics web tool (<https://david.ncifcrf.gov/>) and the KEGG pathway database [45]. A network illustrating all differentially expressed miRNAs from the various comparisons was created using Cytoscape [46].
