# Supplementary figures and images for: Seasonal influence on miRNA expression dynamics of extracellular vesicles in equine follicular fluid
Source: J Anim Sci Biotechnol. 2024 Oct 9;15:137. doi: 10.1186/s40104-024-01097-2 (PMC11462823; doi:10.1186/s40104-024-01097-2)

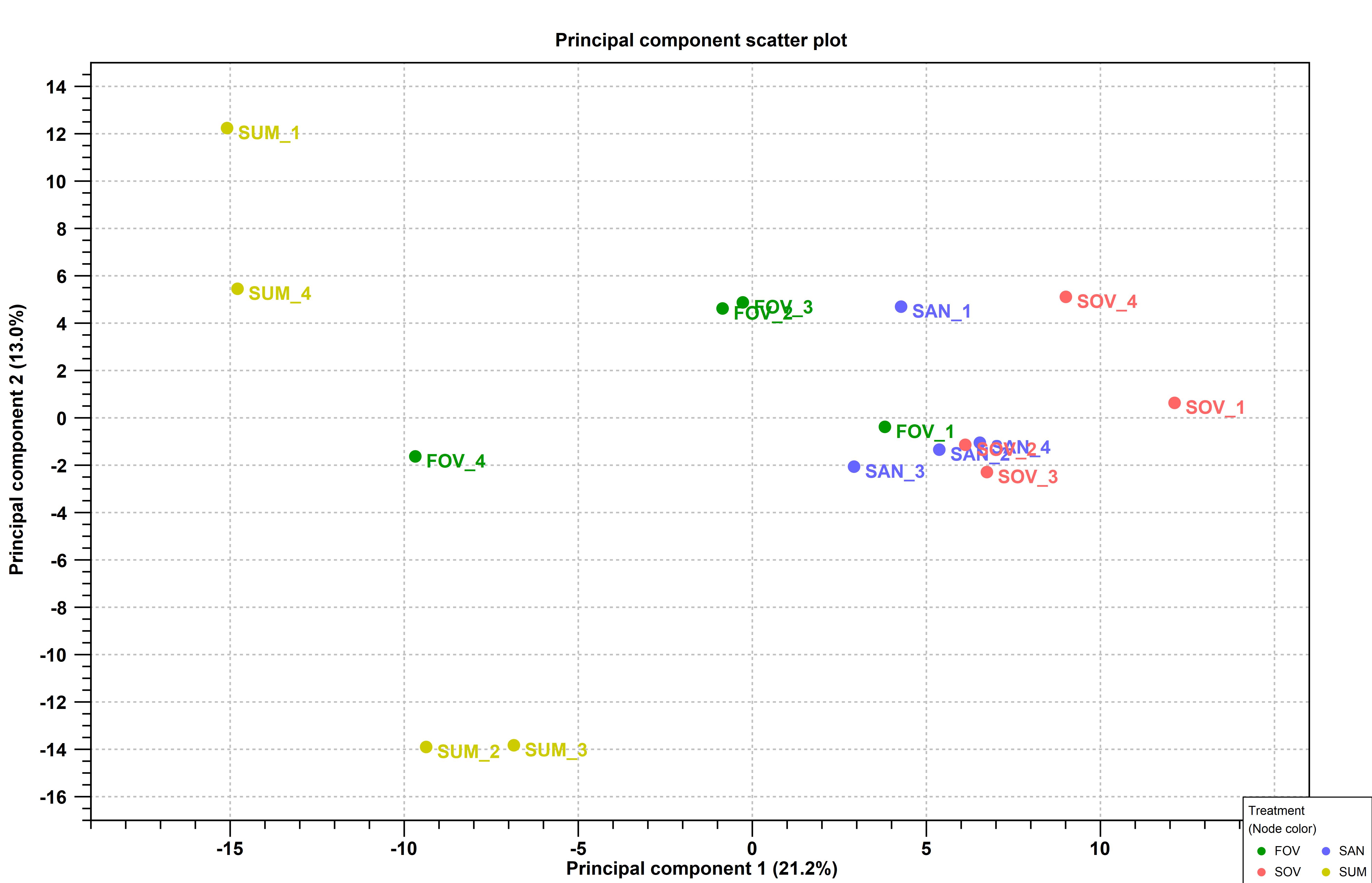

Supplement: Supplementary file 4 — Additional file 4. Principal component analysis: P1/P2. [file 40104_2024_1097_MOESM4_ESM.jpg]
